# Supplementary material for: Primordial germ cells experience increasing physical confinement and DNA damage during migration in the mouse embryo
Source: Sci Adv. 2026 Mar 18;12(12):eaec7919. doi: 10.1126/sciadv.aec7919 (PMC12998503; doi:10.1126/sciadv.aec7919)
Supplement: Supplementary file 1 — Figs. S1 to S6 Table S1 Legends for movies S1 to S7 [file sciadv.aec7919_sm.pdf]

Science Advances
AAAS

Supplementary Materials for

Primordial germ cells experience increasing physical confinement and DNA damage during migration in the mouse embryo

Katharine Goodwin *et al.*

Corresponding author: Katie McDole, kmcdole@mrc-lmb.cam.ac.uk;
Katharine Goodwin, kgoodwin@mrc-lmb.cam.ac.uk

Sci. Adv. **12**, eaec7919 (2026)
DOI: 10.1126/sciadv.aec7919

The PDF file includes:

Figs. S1 to S6
Table S1
Legends for movies S1 to S7

Other Supplementary Material for this manuscript includes the following:

Movies S1 to S7

Figure S1. Cytoskeleton and cell adhesion in migrating PGCs

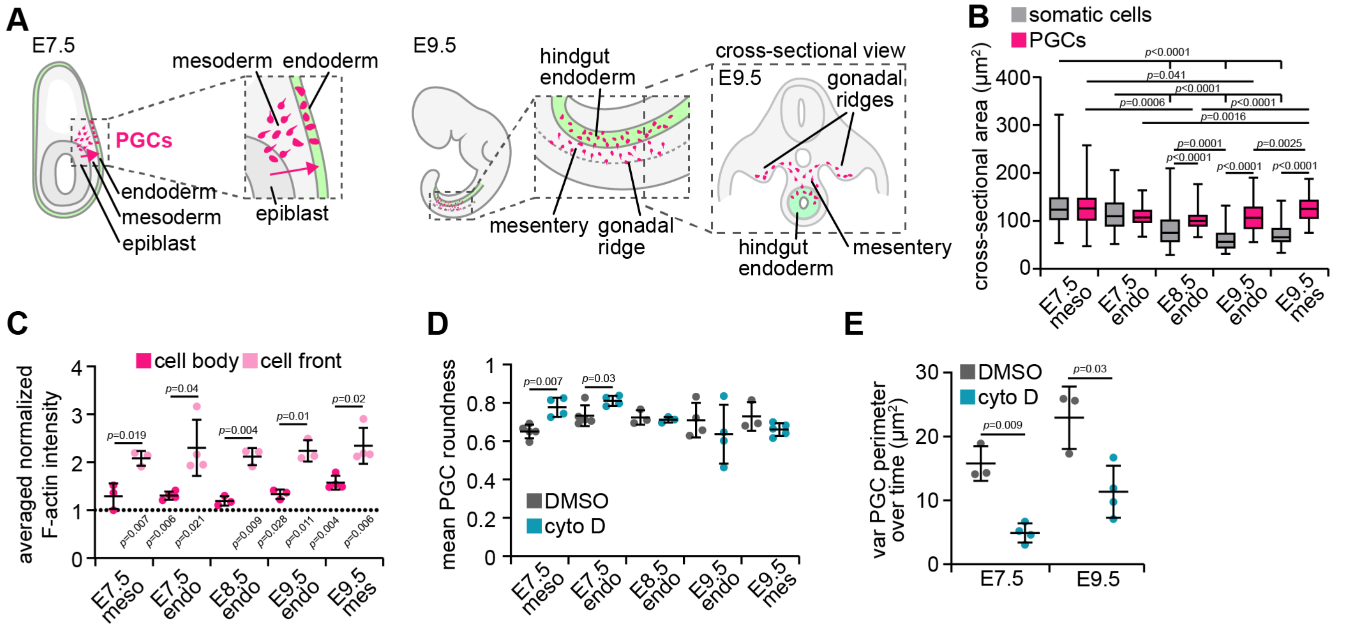

Figure S1. Cytoskeleton and cell adhesion in migrating PGCs

(**A**) Schematics showing zoomed-in views of regions that PGCs migrate through at E7.5 and E9.5.

(**B**) Cross-sectional areas of PGCs and somatic cells from E7.5 to E9.5 in the mesoderm (meso), endoderm (endo), and mesentery (mes).

(**C**) F-actin intensity at cell fronts and cell bodies normalized to neighbouring somatic cells in PGCs from E7.5 to E9.5 and in the mesoderm, endoderm, and mesentery. Averaged values for each embryo are shown. P-values for pairwise comparisons using two-sided t-test are shown above the data points, while those for one-sample t-tests comparing data points to 1 (i.e., equal to somatic cell intensity) are shown below the data points.

(**D**) Average values for each embryo or explants of PGC roundness at each stage and in each tissue in cytochalasin D (1 µg/ml) and DMSO control-treated embryos.

(**E**) Average values for each embryo or explant of variance in PGC perimeter over time, indicating how much PGCs are changing shape, in timelapses of cytochalasin D (1 µg/ml) and DMSO control-treated E7.5 embryos and E9.5 explants.

Figure S2. ECM around migrating PGCs.

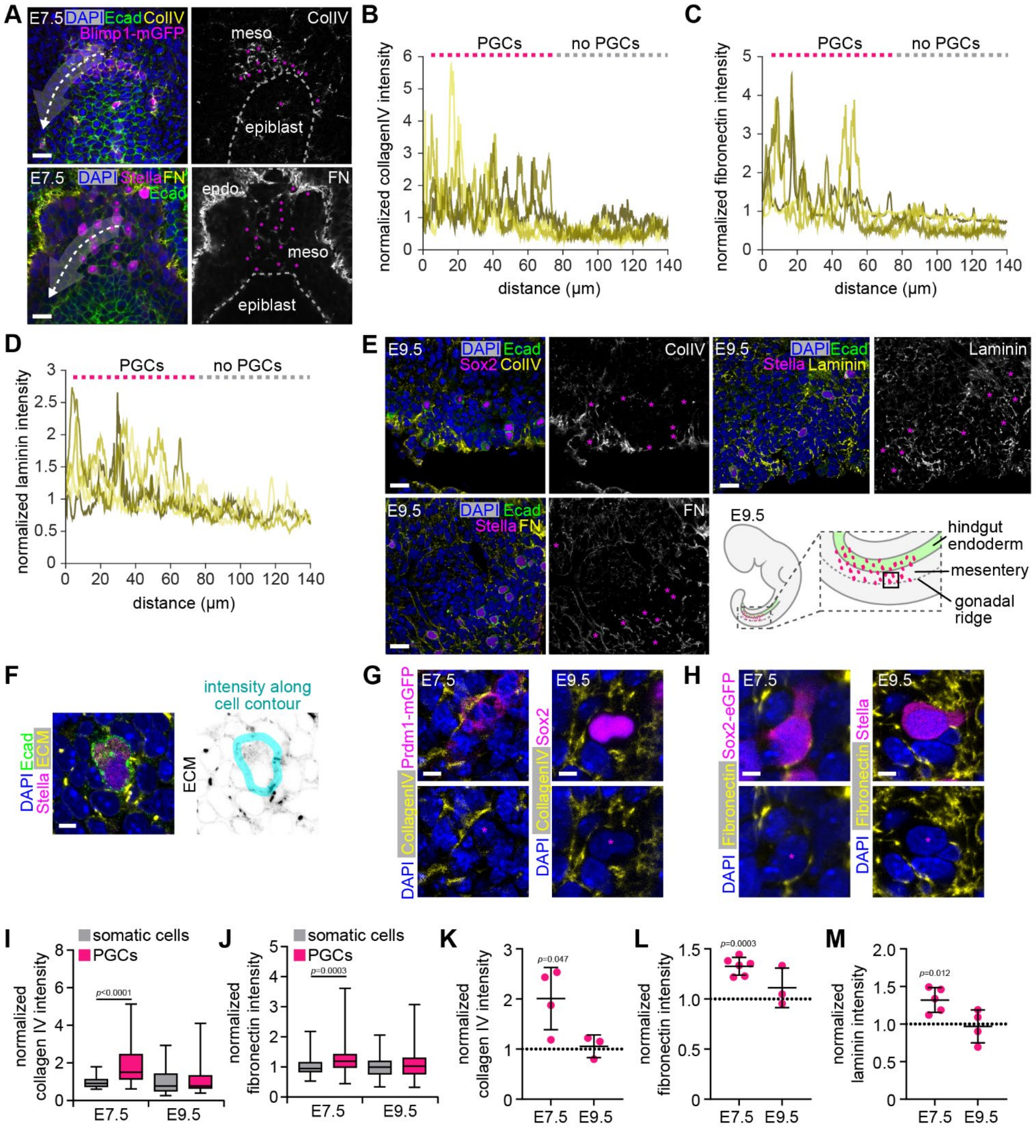

Figure S2. ECM around migrating PGCs

(**A**) Maximum intensity projections of the posterior regions of E7.5 embryos stained for the ECM components collagen IV or fibronectin, the PGC markers Stella or GFP in Sox2-eGFP embryos, Ecad, and DAPI. PGCs are indicated with magenta asterisks. Arrows indicate where intensity profiles in B-D were measured.

(**B-D**) Fluorescence intensity profiles from individual embryos of collagen IV (B), fibronectin (C), and laminin (D) in PGC-containing and adjacent regions of the mesoderm.

(**E**) E9.5 hindguts stained for the ECM components laminin, collagen IV (ColIV) or fibronectin (FN), the PGC markers Stella or Sox2, Ecad, and DAPI. PGCs are indicated with magenta asterisks. Schematic indicates where images were taken.

(**F**) Zoomed-in image of a PGC and the surrounding ECM. Contour indicates where ECM immunofluorescence intensity was measured.

(**G**) PGCs in E7.5 embryos and E9.5 hindguts stained for collagen IV, GFP in Prdm1-mGFP embryos or Sox2 in WT, and DAPI. Magenta asterisks indicate PGCs.

(**H**) PGCs in E7.5 embryos and E9.5 hindguts stained for fibronectin, GFP in Sox2-GFP embryos or Stella in WT, and DAPI. Magenta asterisks indicate PGCs.

(**I-J**) Immunofluorescence intensity of collagen IV (I) and fibronectin (J) along PGC and somatic cell contours, normalized to the mean intensity around somatic cells in each embryo.

(**K-M**) Collagen IV (K), fibronectin (L), and laminin (M) immunofluorescence intensity around PGCs normalized to somatic cells and averaged for each embryo at E7.5 and E9.5. Scale bars 25 µm in (a-b), 5 µm in (c-e).

Figure S3. Laminin reporter mouse and integrin expression during PGC migration

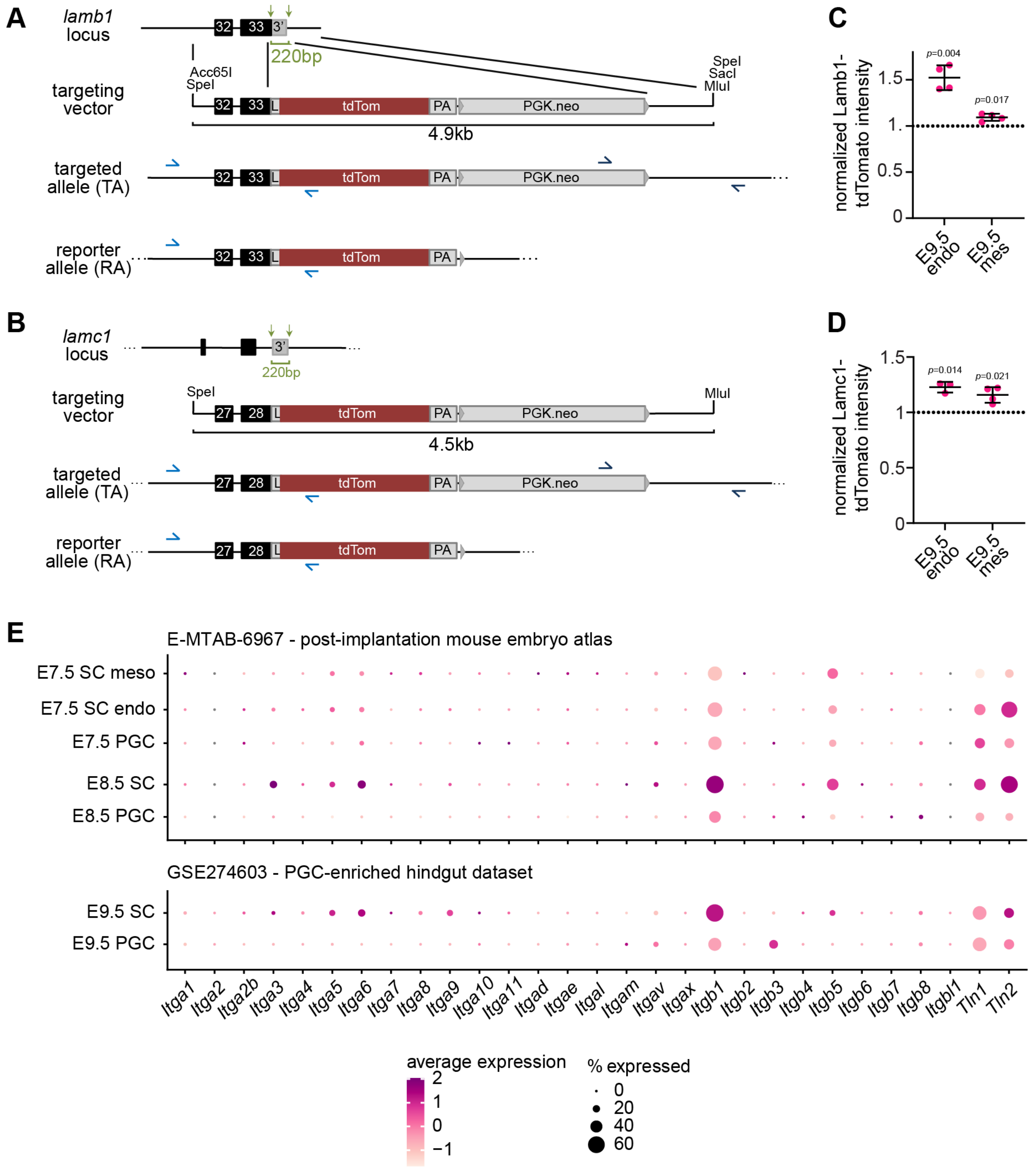

Figure S3. Laminin reporter mouse and integrin expression during PGC Migration

(**A-B**) Targeting strategy using site directed homologous recombination for generation of Lamb1-tdTomato (A) and Lamc1-tdTomato (B) mice (see Methods; black: exons; 3': endogenous 3' UTR; PA: PolyA; L: linker; triangles: loxP sites; green arrows: TALEN binding sites; blue arrows: screening primers).

(**C-D**) Lamb1-tdTomato (M) and Lamc1-tdTomato (N) live reporter intensity around PGCs normalized to somatic cells and averaged for each embryo.

(**E**) Average expression and percentage of cells expressing integrin and talin genes at all stages of PGC migration, in both PGCs and surrounding somatic cells from published single cell RNA-seq datasets.

Figure S4. Changes in intercellular spaces and stiffness in tissues along PGC migratory path and their effect on migrating PGCs

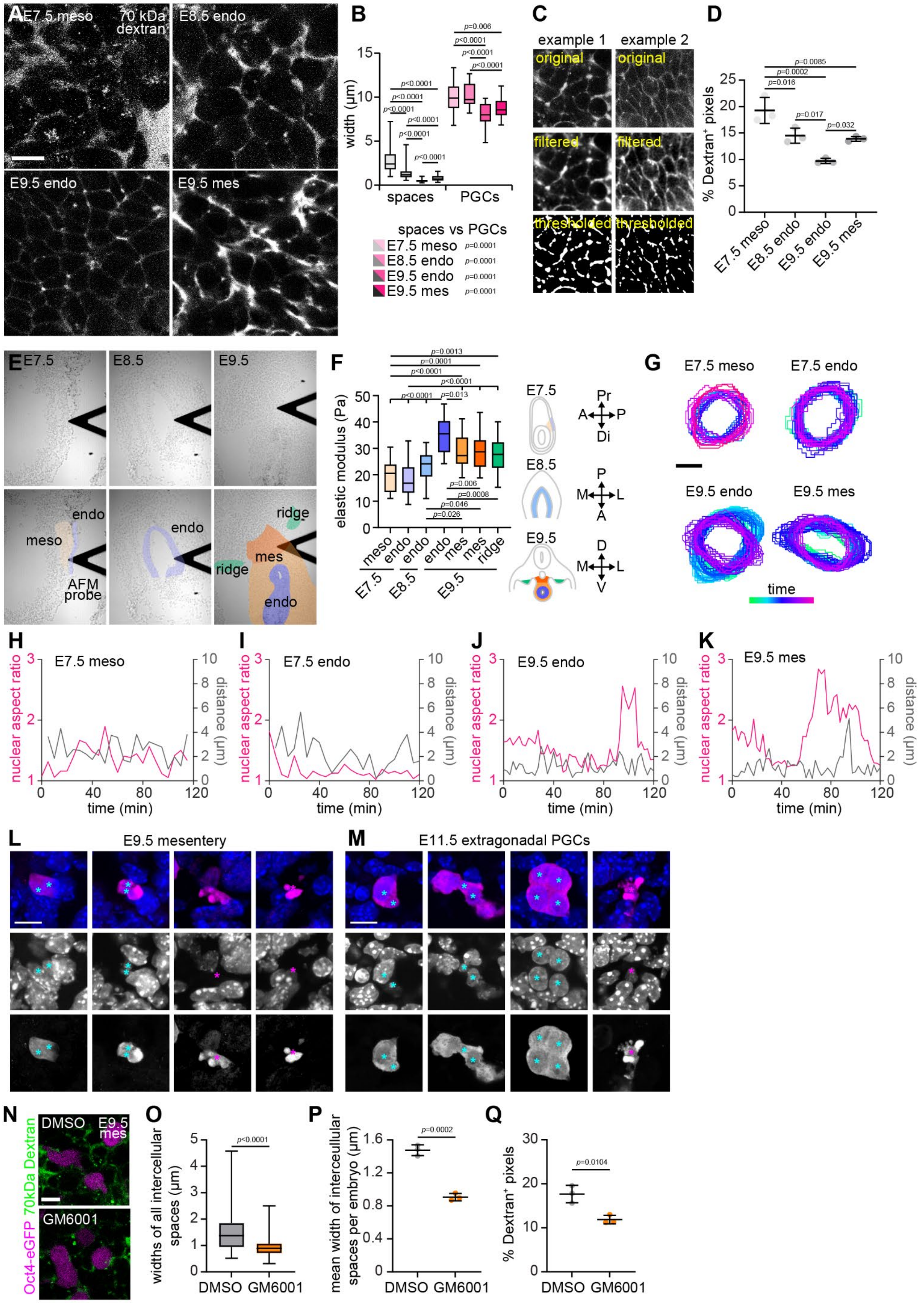

Figure S4. Changes in intercellular spaces and stiffness in tissues along PGC migratory path and their effect on migrating PGCs

(**A**) Snapshots of live E7.5 and E8.5 embryos and E9.5 hindgut incubated with 70 kDa dextran.

(**B**) Widths of all intercellular spaces measured (using Dextran signal) and all PGCs.

(**C**) Two example images of live embryos or explants incubated with 70 kDa dextran, showing originals, filtered images, and final thresholded images used for analysis of intercellular spaces in (D).

(**D**) Percentage of dextran-positive pixels, as a measurement of relative intercellular space, from E7.5 to E9.5 and in each tissue of interest.

(**E**) Brightfield images of tissue sections used for AFM, with the annotated version provided below the originals.

(**F**) All measurements of elastic modulus of tissues around migrating PGCs from E7.5 to E9.5, as indicated by the schematics.

(**G**) Cell contours over time from timelapses of E7.5 embryos and E9.5 explants of PGCs migrating in the mesoderm and endoderm at E7.5 and the endoderm and mesentery at E9.5.

(**H-K**) Aspect ratio and distance travelled of PGC nuclei over the previous 5 minutes during migration in the mesoderm (H) and endoderm (I) at E7.5 and in the endoderm (J) and mesentery (K) at E9.5.

(**L-M**) Examples of migrating PGCs in the mesentery at E9.5 (L) and extragonadal PGCs at E11.5 (M) with evidence of nuclear abnormalities or cell rupture.

(**N**) Hindguts expressing OCT4-eGFP, treated with GM6001 or DMSO control and incubated with 70 kDa dextran to show intercellular spaces.

(**O**) Widths of intercellular spaces in DMSO controls and GM6001-treated hindguts.

(**P**) Mean widths of intercellular spaces in DMSO controls and GM6001-treated hindguts.

(**Q**) Percentage of dextran-positive pixels, as a measurement of relative intercellular space, in the mesentery of GM6001-treated explants and DMSO controls.

Scale bars 10 µm.

Figure S5. Migrating PGCs exhibit increasing evidence of DNA damage

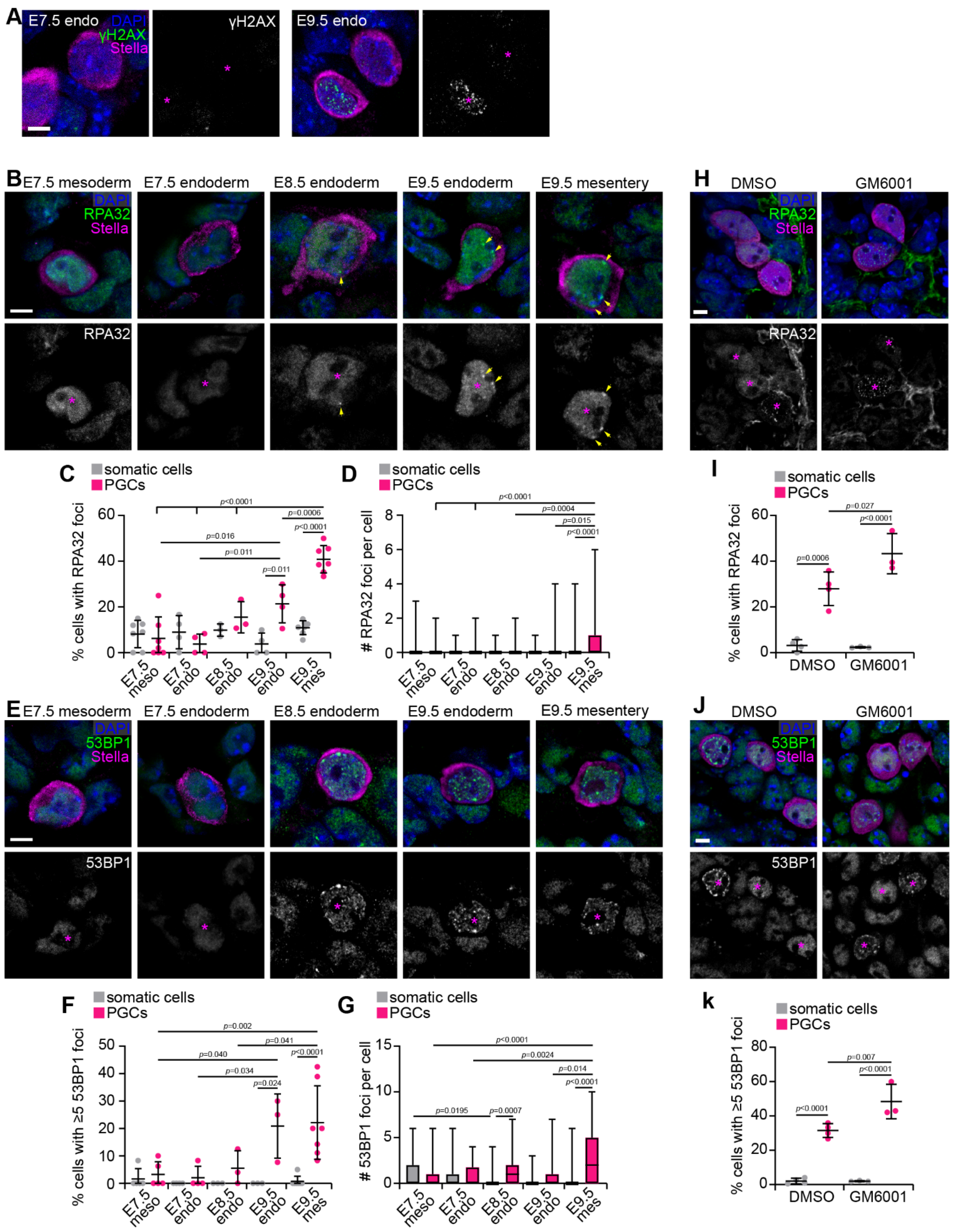

Figure S5. Migrating PGCs exhibit increasing evidence of DNA damage

(**A**) Sections of E7.5 and E9.5 embryos stained for γH2AX, Stella and DAPI for tissues/stages not shown in Fig 5. Asterisks indicate PGCs.

(**B**) Sections of E7.5, E8.5, and E9.5 embryos stained for the DNA damage marker RPA32 and the PGC marker Stella and counterstained with DAPI. PGCs are indicated with pink asterisks. Yellow arrows indicate RPA32 foci.

(**C**) Percentage of cells with RPA32 foci in PGCs and in surrounding somatic cells in the relevant tissues at each developmental stage.

(**D**) Number of RPA32 foci per cell in the mesoderm (meso), endoderm (endo), and mesentery (mes) at E7.5, E8.5, and E9.5.

(**E**) Sections of E7.5, E8.5, and E9.5 embryos stained for the DNA damage marker 53BP1 and the PGC marker Stella and counterstained with DAPI. PGCs are indicated with pink asterisks.

(**F**) Percentage of cells with ≥5 53BP1 foci in PGCs and in surrounding somatic cells in the relevant tissues at each developmental stage.

(**G**) Number of 53BP1 foci per cell in the mesoderm, endoderm, and mesentery at E7.5, E8.5, and E9.5.

(**H**) Sections of E9.5 hindguts cultured with DMSO or GM6001 and stained for RPA32, Stella and DAPI. Asterisks indicate PGCs.

(**I**) Percentage of cells with RPA32 foci in PGCs and in surrounding somatic cells in E9.5 hindgut cultured with DMSO or GM6001.

(**J**) Sections of E9.5 hindguts cultured with DMSO or GM6001 and stained for 53BP1, Stella and DAPI. Asterisks indicate PGCs.

(**K**) Percentage of cells with ≥5 53BP1 foci in PGCs and in surrounding somatic cells in E9.5 hindgut cultured with DMSO or GM6001.

Scale bars 5 μm.

Figure S6. Nuclear lamina and nuclear morphology in migrating PGCs

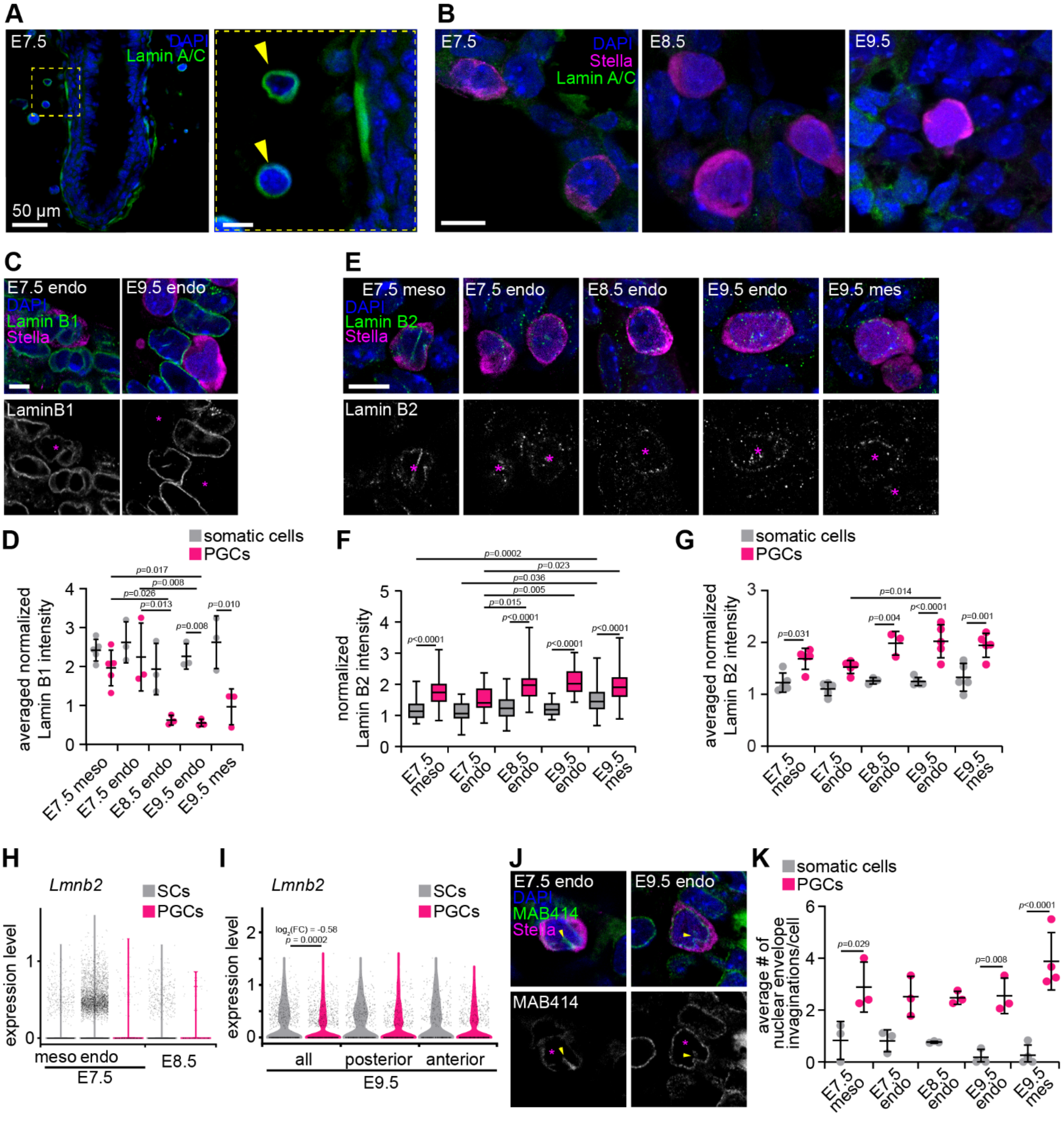

Figure S6. Nuclear lamina and nuclear morphology in migrating PGCs

(**A**) Section of an E7.5 embryo stained for Lamin A/C and DAPI. Lamin A/C is expressed in maternal decidual cells (yellow arrowheads), but not in embryonic cells.

(**B**) Sections of E7.5, E8.5, and E9.5 embryos stained for Lamin A/C, Stella, and DAPI.

(**C**) Sections of E7.5 and E9.5 embryos stained for Lamin B1, Stella and DAPI for tissues/stages not shown in Fig. 5.

(**D**) Immunofluorescence intensity of Lamin B1 around the nucleus of PGCs in the relevant tissues at each developmental stage, normalized to mean fluorescence intensity and averaged for each embryo.

(**E**) Sections of E7.5, E8.5, and E9.5 embryos stained for Lamin B2 and the PGC marker Stella and counterstained with DAPI. PGCs are indicated with pink asterisks.

(**F**) Immunofluorescence intensity of Lamin B2 around the nucleus of PGCs and surrounding somatic cells in the relevant tissues at each developmental stage, normalized to mean fluorescence intensity.

(**G**) Immunofluorescence intensity of Lamin B2 around the nucleus of PGCs in the relevant tissues at each developmental stage, normalized to mean fluorescence intensity and averaged for each embryo.

(**H-I**) Expression levels, $\log_2$ fold changes (FC) and corresponding adjusted p values of *Lmnb2* in PGCs and surrounding somatic cells from single cell RNA-seq datasets of E7.5 to E8.5 embryos (H) and of E9.5 hindguts (I). For E9.5, all PGCs together are shown, along with leading migrants in anterior dissections and lagging migrants in the posterior dissections.

(**L**) Sections of E7.5 and E9.5 embryos stained for MAB414, Stella and DAPI for tissues/stages not shown in Fig. 5. Yellow arrows indicate invaginations of the nuclear envelope.

(**M**) Average number of nuclear envelope invaginations per cell.

Scale bars 10 µm unless otherwise indicated.

				individual measurements			
figure	panel	group	n embryos	type	n	error bars/box plot elements	statistical tests
1	E	E7.5 mesoderm	2	somatic cell protrusions	237	box: 25th to 75th percentiles, line: median; whiskers: min and max	One-way ANOVA with Tukey's multiple comparisons test. Only comparisons between PGCs and somatic cells at the same stage and between somatic cells or PGCs at different developmental stages are shown .
			3	PGC protrusions	169		
		E9.5 mesentery	2	somatic cell protrusions	205		
			2	PGC protrusions	150		
	F	E7.5 mesoderm	2	somatic cells	88		
			3	PGCs	49		
		E7.5 endoderm	3	somatic cells	73		
			3	PGCs	73		
		E8.5 endoderm	2	somatic cells	100		
			2	PGCs	100		
		E9.5 endoderm	3	somatic cells	66		
			3	PGCs	66		
		E9.5 mesentery	2	somatic cells	83		
			2	PGCs	84		
	I	E7.5 mesoderm	3	PGCs	21	N/A	Paired t test
		E7.5 endoderm	4		24		
		E8.5 endoderm	3		30		
		E9.5 endoderm	3		18		
		E9.5 mesentery	4		26		
	M-O	E7.5 mesoderm	5	DMSO	52	(M-N) error bars indicate s.d., (O) box: 25th to 75th percentiles, line: median; whiskers: min and max	(M) 2 way ANOVA with Tukey's multiple comparisons test, (N) Welch's t-test to compare treated samples to stage-matched DMSO controls, (O) Komogorov-Smirnov test to compare treated samples to stage-matched DMSO controls
			4	cytochalasin D	38		
		E7.5 endoderm	5	DMSO	67		
			4	cytochalasin D	48		
		E8.5 endoderm	3	DMSO	87		
			3	cytochalasin D	66		
		E9.5 endoderm	5	DMSO	28		
			4	cytochalasin D	30		
		E9.5 mesentery	4	DMSO	64		
			5	cytochalasin D	47		
	Q	E7.5	4	DMSO	46	box: 25th to 75th percentiles, line: median; whiskers: min and max	Welch's t-test to compare treated samples to stage-matched DMSO controls
			4	cytochalasin D	34		
		E9.5	3	DMSO	155		
			4	cytochalasin D	86		
2	B	collagen IV	4	N/A	N/A	s.d.	One-sample t-test comparing data points to 1 (i.e., equal intensity between regions)
		fibronectin	4				
		laminin	3				
	D	E7.5 mesoderm	5	somatic cells	57	box: 25th to 75th percentiles, line: median; whiskers: min and max	Welch's t-test for each stage
				PGCs	57		
		E9.5 mesentery	4	somatic cells	57		
				PGCs	57		
	H	E9.5 endoderm	4	somatic cells	80	box: 25th to 75th percentiles, line: median; whiskers: min and max	Welch's t-test for each tissue
				PGCs	80		
		E9.5 mesentery	4	somatic cells	60		
				PGCs	60		
		E9.5 endoderm	3	somatic cells	31		
				PGCs	31		
		E9.5 mesentery	4	somatic cells	81		
				PGCs	81		
3	A, B	collagen IV, E7.5	4	measurements	20	s.d.	One-way Brown-Forsythe and Welch ANOVA and Dunnett's T3 test for multiple comparisons
		collagen IV, E8.5	8	measurements	40		
		collagen IV, E9.5	5	measurements	25		
		fibronectin, E7.5	10	measurements	50		
		fibronectin, E8.5	6	measurements	30		
		fibronectin, E9.5	3	measurements	15		
		laminin, E7.5	5	measurements	25		
		laminin, E8.5	4	measurements	20		
		laminin, E9.5	6	measurements	30		
	F	E7.5	6	PGCs	19	s.d.	Two-sided t-test
		E9.5	6		8		
	H	E7.5	1	PGCs	22	N/A	N/A
	J	E9.5	3	PGCs	4	N/A	Note: one cell with three protrusions
	L	E9.5	4	PGCs	10	s.d.	Simple linear regression
4	A	E7.5 mesoderm	4	intercellular spaces	107	s.d.	Brown-Forsythe and Welch ANOVA and Dunnett's T3 test
				PGCs	36		
		E8.5 endoderm	4	intercellular spaces	147		
				PGCs	49		
		E9.5 endoderm	4	intercellular spaces	120		
				PGCs	40		
		E9.5 mesentery	4	intercellular spaces	120		
				PGCs	40		
	B	E7.5 mesoderm	4	AFM measurements	22	s.d.	Ordinary one-way ANOVA with Tukey's multiple comparisons test
		E7.5 endoderm	4		20		
		E8.5 endoderm	4		23		
		E9.5 endoderm	4		19		
		E9.5 mesentery - close	5		26		
		E9.5 mesentery - far	5		27		
		E9.5 gonadal ridge	5		26		
	G	E7.5 mesoderm	1	PGC nuclei	15	n/a	Paired t test for comparisons between matched measurements and one-way ANOVA with Tukey's multiple comparisons test for comparisons between stages
		E7.5 entering			10		
		E7.5 endoderm			15		
		E9.5 endoderm	3		13		
		E9.5 exiting			10		
		E9.5 mesentery			13		

	K	DMSO	4	PGCs	219	s.d.	Two-way ANOVA with Šídák's multiple comparisons test
		GM6001	4		296		
5	B	E7.5 mesoderm	3	somatic cells	28	s.d.	One-way ANOVA with Tukey's multiple comparisons test. Only comparisons between PGCs and somatic cells at the same stage and between PGCs or somatic cells at different developmental stages are shown.
				PGCs	28		
		E7.5 endoderm	2	somatic cells	18		
				PGCs	18		
		E8.5 endoderm	3	somatic cells	67		
				PGCs	67		
		E9.5 endoderm	3	somatic cells	42		
				PGCs	42		
		E9.5 mesentery	6	somatic cells	123		
				PGCs	123		
	D	DMSO	3	somatic cells	1277	s.d.	One-way ANOVA and Tukey's multiple comparisons test. Only comparisons between PGCs and somatic cells within each treatment or between treatments within each cell type are shown.
				PGCs	92		
		GM6001	4	somatic cells	1355		
				PGCs	75		
	H	E7.5 mesoderm	5	somatic cells	60	box: 25th to 75th percentiles, line: median; whiskers: min and max	Krusal-Wallis test with Dunn's Multiple Comparisons Test. Only comparisons between PGCs and somatic cells at the same stage and between PGCs or somatic cells at different developmental stages are shown.
				PGCs	50		
		E7.5 endoderm	3	somatic cells	25		
				PGCs	23		
		E8.5 endoderm	3	somatic cells	53		
				PGCs	53		
		E9.5 endoderm	3	somatic cells	35		
				PGCs	31		
		E9.5 mesentery	3	somatic cells	30		
				PGCs	30		
	J	E7.5 mesoderm	3	somatic cells	21	box: 25th to 75th percentiles, line: median; whiskers: min and max	Krusal-Wallis test with Dunn's Multiple Comparisons Test. Only comparisons between PGCs and somatic cells at the same stage and between PGCs or somatic cells at different developmental stages are shown.
				PGCs	21		
		E7.5 endoderm	3	somatic cells	29		
				PGCs	29		
		E8.5 endoderm	3	somatic cells	33		
				PGCs	33		
		E9.5 endoderm	3	somatic cells	45		
				PGCs	45		
		E9.5 mesentery	4	somatic cells	22		
				PGCs	22		
S1	B	E7.5 mesoderm	2	somatic cells	88	box: 25th to 75th percentiles, line: median; whiskers: min and max	One-way ANOVA with Tukey's multiple comparisons test. Only comparisons between PGCs and somatic cells at the same stage and between somatic cells or PGCs at different developmental stages are shown .
			3	PGCs	49		
		E7.5 endoderm	3	somatic cells	73		
			3	PGCs	73		
		E8.5 endoderm	2	somatic cells	100		
			2	PGCs	100		
		E9.5 endoderm	3	somatic cells	66		
			3	PGCs	66		
		E9.5 mesentery	2	somatic cells	83		
			2	PGCs	84		
	C	E7.5 mesoderm	3	PGCs	21	s.d.	Two-sided t-test for pairwise comparisons, one-sample t-tests comparing data points to 1 (i.e., equal to somatic cell intensity)
		E7.5 endoderm	4		24		
		E8.5 endoderm	3		30		
		E9.5 endoderm	3		18		
		E9.5 mesentery	4		26		
	D	E7.5 mesoderm	5	DMSO	52	error bars indicate s.d.	Welch's t-test to compare treated samples to stage-matched DMSO controls
			4	cytochalasin D	38		
		E7.5 endoderm	5	DMSO	67		
			4	cytochalasin D	48		
		E8.5 endoderm	3	DMSO	87		
			3	cytochalasin D	66		
		E9.5 endoderm	5	DMSO	28		
			4	cytochalasin D	30		
		E9.5 mesentery	4	DMSO	64		
			5	cytochalasin D	47		
	E	E7.5	4	DMSO	46	error bars indicate s.d.	Welch's t-test to compare treated samples to stage-matched DMSO controls
			4	cytochalasin D	34		
		E9.5	3	DMSO	155		
			4	cytochalasin D	86		
S2	B	collagen IV	4	intensity profiles	n/a	n/a	n/a
	C	fibronectin	4				
	D	laminin	5				
	I, K	E7.5 mesoderm	4	somatic cells	38	(I, J) box: 25th to 75th percentiles, line: median; whiskers: min and max, (K, L) s.d.	(F-G) Welch's t-test for each stage, (H-I) One-sample t-tests comparing data points to 1 (i.e., equal to somatic cell intensity)
				PGCs	38		
		E9.5 mesentery	3	somatic cells	42		
				PGCs	42		
	J, L	E7.5 mesoderm	6	somatic cells	62		
				PGCs	62		
		E9.5 mesentery	3	somatic cells	42		
				PGCs	42		
	M	E7.5 mesoderm	5	somatic cells	57	s.d.	One-sample t-tests comparing data points to 1 (i.e., equal to somatic cell intensity)
				PGCs	57		
		E9.5 mesentery	4	somatic cells	57		
				PGCs	57		
S3	C	E9.5 endoderm	4	somatic cells	80	s.d.	One-sample t-tests comparing data points to 1 (i.e., equal to somatic cell intensity)
				PGCs	80		
		E9.5 mesentery	4	somatic cells	60		
				PGCs	60		

	D	E9.5 endoderm	3	somatic cells	31	s.d.	One-sample t-tests comparing data points to 1 (i.e., equal to somatic cell intensity)
				PGCs	31		
		E9.5 mesentery	4	somatic cells	81		
				PGCs	81		
S4	B	E7.5 mesoderm	4	intercellular spaces	107	box: 25th to 75th percentiles, line: median; whiskers: min and max	One-way Brown-Forsythe and Welch ANOVA and Dunnett's T3 test for multiple comparisons, focusing on differences between intercellular spaces at each stage and between intercellular spaces and PGCs within each stage
				PGCs	36		
		E8.5 endoderm	4	intercellular spaces	147		
				PGCs	49		
		E9.5 endoderm	4	intercellular spaces	120		
				PGCs	40		
		E9.5 mesentery	4	intercellular spaces	120		
				PGCs	40		
	D	E7.5 endoderm	3	n/a		s.d.	One-way ANOVA with Tukey's multiple comparisons test
		E8.5 endoderm	3				
		E9.5 endoderm	3				
		E9.5 mesentery	3				
	F	E7.5 mesoderm	4	AFM measurements	22	box: 25th to 75th percentiles, line: median; whiskers: min and max	One-way ANOVA with Tukey's multiple comparisons test
		E7.5 endoderm	4		20		
		E8.5 endoderm	4		23		
		E9.5 endoderm	4		19		
		E9.5 mesentery - close	5		26		
		E9.5 mesentery - far	5		27		
		E9.5 gonadal ridge	5		26		
	O-Q	DMSO	3	intercellular spaces	60	s.d.	(O) Mann-Whitney test, (P-Q) two-sided t-test
		GM6001	3		60		
S5	C, D	E7.5 mesoderm	7	somatic cells	85	(c) s.d.	(C) One-way ANOVA with Tukey's multiple comparisons test, (D) Kruskal-Wallis test with Dunn's multiple comparisons test. Only comparisons between PGCs and somatic cells at the same stage and between PGCs or somatic cells at different developmental stages are shown
				PGCs	82		
		E7.5 endoderm	4	somatic cells	64		
				PGCs	64		
		E8.5 endoderm	3	somatic cells	66		
				PGCs	66		
		E9.5 endoderm	4	somatic cells	63		
				PGCs	51		
		E9.5 mesentery	7	somatic cells	189		
				PGCs	96		
	F, G	E7.5 mesoderm	5	somatic cells	97	(f) s.d.	(F) One-way ANOVA with Tukey's multiple comparisons test, (G) Kruskal-Wallis test with Dunn's multiple comparisons test. Only comparisons between PGCs and somatic cells at the same stage and between PGCs or somatic cells at different developmental stages are shown
				PGCs	97		
		E7.5 endoderm	5	somatic cells	64		
				PGCs	64		
		E8.5 endoderm	3	somatic cells	63		
				PGCs	63		
		E9.5 endoderm	3	somatic cells	31		
				PGCs	31		
		E9.5 mesentery	4	somatic cells	123		
				PGCs	111		
	I	DMSO	4	somatic cells	2782	s.d.	One-way ANOVA with Tukey's multiple comparisons test. Only comparisons between PGCs and somatic cells within each treatment or between treatments within each cell type are shown.
				PGCs	161		
		GM6001	3	somatic cells	1226		
				PGCs	153		
	K	DMSO	4	somatic cells	1317	s.d.	One-way ANOVA with Tukey's multiple comparisons test. Only comparisons between PGCs and somatic cells within each treatment or between treatments within each cell type are shown.
				PGCs	76		
		GM6001	3	somatic cells	1226		
				PGCs	153		
S6	D	E7.5 mesoderm	5	somatic cells	60	s.d.	One-way ANOVA with Tukey's multiple comparisons test. Only comparisons between PGCs and somatic cells at the same stage and between PGCs or somatic cells at different developmental stages are shown.
				PGCs	50		
		E7.5 endoderm	3	somatic cells	25		
				PGCs	23		
		E8.5 endoderm	3	somatic cells	53		
				PGCs	53		
		E9.5 endoderm	3	somatic cells	35		
				PGCs	31		
		E9.5 mesentery	3	somatic cells	30		
				PGCs	30		
	F, G	E7.5 mesoderm	5	somatic cells	48	(F) box: 25th to 75th percentiles, line: median; whiskers: min and max, (G) s.d.	(F) Krusal-Wallis test with Dunn's multiple vomparisons test, (FG One-way ANOVA with Tukey's multiple comparisons test. Only comparisons between PGCs and somatic cells at the same stage and between PGCs or somatic cells at different developmental stages are shown.
				PGCs	48		
		E7.5 endoderm	5	somatic cells	22		
				PGCs	22		
		E8.5 endoderm	3	somatic cells	54		
				PGCs	54		
		E9.5 endoderm	5	somatic cells	32		
				PGCs	29		
		E9.5 mesentery	5	somatic cells	132		
				PGCs	122		
	M	E7.5 mesoderm	3	somatic cells	21	s.d.	One-way ANOVA with Tukey's multiple comparisons test. Only comparisons between PGCs and somatic cells at the same stage and between PGCs or somatic cells at different developmental stages are shown.
				PGCs	21		
		E7.5 endoderm	3	somatic cells	29		
				PGCs	29		
		E8.5 endoderm	3	somatic cells	33		
				PGCs	33		
		E9.5 endoderm	3	somatic cells	45		
				PGCs	45		
		E9.5 mesentery	4	somatic cells	22		
				PGCs	22		

Table S1. Experimental numbers and statistical test details for all figures.

Movie S1. Protrusive migration of a PGC in the mesentery of an E9.5 hindgut explant expressing Sox2-eGFP and mKate2-nls.

Movie S2. PGC migration coincides with the appearance of laminin in an E7.5 embryo expressing Sox2-eGFP and Lamb1-tdTomato.

Movie S3. F-actin-rich protrusion formation by a PGC in the hindgut endoderm of an E9.5 hindgut explant expressing Sox2-eGFP and LifeAct-RFP. Arrows point to locations of these protrusions at different points within the movie.

Movie S4. Dynamic and long-lived protrusion into the mesentery made by a PGC in the hindgut endoderm of an E9.5 hindgut explant expressing Oct4-eGFP and Lamc1-tdTomato.

Movie S5. Protrusion extension, widening, and PGC exit from the hindgut endoderm in an E9.5 hindgut explant expressing Sox2-eGFP and mKate2-nls.

Movie S6. PGC exiting the hindgut endoderm and immediately fragmenting in an E9.5 hindgut explant expressing Sox2-eGFP and mKate2-nls.

Movie S7. DMSO control and GM6001-treated hindgut explants expressing Sox2-eGFP showing normal migration in controls and fast migration followed by cell rupture when MMP activity is inhibited and confinement is increased.
